# Trichoderma-Mediated ZnO Nanoparticles and Their Antibiofilm and Antibacterial Activities

**DOI:** 10.3390/jof9020133

**Published:** 2023-01-18

**Authors:** Balagangadharaswamy Shobha, Bagepalli Shivaram Ashwini, Mohammed Ghazwani, Umme Hani, Banan Atwah, Maryam S. Alhumaidi, Sumanth Basavaraju, Srinivas Chowdappa, Tekupalli Ravikiran, Shadma Wahab, Wasim Ahmad, Thimappa Ramachandrappa Lakshmeesha, Mohammad Azam Ansari

**Affiliations:** 1 Department of Microbiology and Biotechnology, Bangalore University, Jnana Bharathi Campus, Bengaluru 560056, India; shobhahonnaganga@gmail.com (B.S.); simplesumanth007@gmail.com (S.B.); csrinivas@bub.ernet.in (S.C.); ravikiran@bub.ernet.in (T.R.); 2 Department of Microbiology, Sri Siddhartha Medical College, Tumkur 572107, India; ashwinibs17@gmail.com; 3 Department of Pharmaceutics, College of Pharmacy, King Khalid University, Abha 62529, Saudi Arabia; myghazwani@kku.edu.sa (M.G.); uahmed@kku.edu.sa (U.H.); 4 Laboratory Medicine Department, Faculty of Applied Medical Sciences, Umm Al-Qura University, Makkah 24382, Saudi Arabia; baatwah@uqu.edu.sa; 5 Department of Biology, College of Science, University of Hafr Al Batin, Hafr Al Batin 31991, Saudi Arabia; maryamalhumaidi@uhb.edu.sa; 6 Department of Pharmacognosy, College of Pharmacy, King Khalid University, Abha 61421, Saudi Arabia; sabdulwahab@kku.edu.sa; 7 Department of Pharmacy, Mohammed Al-Mana College for Medical Sciences, Dammam 34222, Saudi Arabia; wasimahmadansari@yahoo.com; 8 Department of Epidemic Disease Research, Institute for Research and Medical Consultations (IRMC), Imam Abdulrahman Bin Faisal University, Dammam 31441, Saudi Arabia

**Keywords:** myconanotechnology, ZnO nanoparticles, nanofabrication, antimicrobial resistance, biofilm, Trichoderma, green synthesis

## Abstract

Antimicrobial resistance is a major global health concern and one of the gravest challenges to humanity today. Antibiotic resistance has been acquired by certain bacterial strains. As a result, new antibacterial drugs are urgently required to combat resistant microorganisms. Species of *Trichoderma* are known to produce a wide range of enzymes and secondary metabolites that can be exploited for the synthesis of nanoparticles. In the present study, *Trichoderma asperellum* was isolated from rhizosphere soil and used for the biosynthesis of ZnO NPs. To examine the antibacterial activity of ZnO NPs against human pathogens, *Escherichia coli* and *Staphylococcus aureus* were used. The obtained antibacterial results show that the biosynthesized ZnO NPs were efficient antibacterial agents against the pathogens *E. coli* and *S. aureus*, with an inhibition zone of 3–9 mm. The ZnO NPs were also effective in the prevention of *S. aureus* biofilm formation and adherence. The current work shows that the MIC dosages of ZnO NPs (25, 50, and 75 μg/mL) have effective antibacterial activity and antibiofilm action against *S. aureus*. As a result, ZnO NPs can be used as a part of combination therapy for drug-resistant *S. aureus* infections, where biofilm development is critical for disease progression.

## 1. Introduction

Antimicrobial resistance is a major worldwide health concern and one of humanity’s most severe threats today [1]. Antibiotic resistance has been acquired by certain bacterial strains. As a result, new antibacterial drugs are urgently required to combat resistant microorganisms [2]. The human body acts as a host to a wide range of microbial communities [3], and the complex interactions that exist between the host and its microbial community play a crucial role in human health and diseases [4].

The facultative non-pathogenic human microflora is dominated by the Gram-negative *E. coli* bacterium. However, some *E. coli* strains have acquired the property to cause urinary tract infections, gastrointestinal illnesses, and central nervous system illnesses in the most resistant human hosts [5]. The bacterium *S. aureus* is a Gram-positive bacterium, and it can grow in both aerobic and anaerobic environments as a facultative bacterium [6]. *S. aureus* is the most common bacterial pathogen and continues to endanger public health [7,8], causing a variety of symptoms in infected people and being found in normal human flora and the environment. *S. aureus* can be isolated from the skin and mucous membranes (most often the nasal parts) of healthy individuals.

Myconanotechnology is a term that combines mycology and nanotechnology [9], and it offers a lot of potential in nanoparticle synthesis [10]. As it is simple to grow fungi in bulk and the extracellular release of enzymes provides an advantage in downstream processing, the production of nanoparticles employing fungi has drawn a lot of attention. Fungi synthesize more proteins than bacteria, leading to increased nanoparticle production. Fungi have been extensively explored due to their characteristics and their rapid and eco-friendly production of metal nanoparticles [11]. It has been observed that the nanoparticles produced by fungi have homogeneous diameters and monodispersity. Nanoparticles are synthesized via a biological approach using intracellular and extracellular mechanisms [12]. Because the intracellular process requires an additional step to obtain pure nanoparticles, the extracellular method is preferable over the intracellular approach [13]. Microorganisms act as reducing and capping agents [14].

Nanotechnology is the study and application of particles with a size range of 1 to 1000 nm [15]. Nanoparticles have unique features due to their small size compared to their bulk counterpart, making them excellent for applications in domains such as electronics, energy, the environment, and health [10]. Physical, chemical, and biological processes can be used to synthesize nanoparticles with desired characteristics, such as size and form [16]. However, because of the high cost and toxicity of the chemicals employed in synthesis, physical and chemical procedures are rarely used. As a result, several studies on the biological or green synthesis of metallic nanoparticles, such as silver, gold, titanium dioxide, iron oxide, magnesium oxide, zinc oxide, copper, and aluminum oxide nanoparticles, have been conducted [17]. Scientists all around the globe have been attracted to zinc oxide nanoparticles (ZnO NPs) because of their therapeutic properties. Pathogenic bacteria can be killed with the use of zinc oxide nanoparticles as an antimicrobial treatment. Several plants and microbes have been reported to synthesize ZnO NPs through the biosynthesis process [18,19]. The antibacterial activity of ZnO NPs could be increased via doping with ions [20]. As the nanoparticles fill the gaps between larger particles and atomic or molecular structures, they are of tremendous scientific interest [21]. Controlling the size and form of nanoparticles to adjust their optical, electronic, and electrical properties is a challenge in nanotechnology. The ideal metal nanoparticles are perfect monodispersed metal nanoparticles [22]. Green synthesis, also known as biosynthesis, is a biological technique of synthesizing ZnO NPs that involves the employment of microorganisms, such as algae, fungus, yeast, bacteria, and plant extracts, as the reducing agents [23]. Despite the benefits of using microbes as reducing and stabilizing agents during the biosynthesis of ZnO NPs, extra caution is necessary due to the toxicity of certain bacteria, as well as incubation concerns.

Plant-growth-promoting microbes (PGPMs) are rhizosphere microorganisms that can colonize the root environment. Bacteria and fungi that can colonize the roots and rhizosphere soil are among the microorganisms that live in this zone. The group of plant-growth-promoting fungi (PGPFs) includes certain *Trichoderma* species that have been identified with plant roots, where they create a symbiotic association or act as endophytes [24]. Plants’ root-driven beneficial activities are mostly determined by their interactions with a wide range of microbial populations in the environment. Weindling was the first to describe the antibiotic synthesis of *Trichoderma* spp. [25]. There are several reports available regarding the *Trichoderma* species compounds proved to have antibacterial properties, volatile compounds (e.g., hydrogen cyanide, ethylene, monoterpenes, and alcohol), and non-volatile compounds (e.g., diketopiperazine, such as gliotoxin and gliovirin, and peptaibols) [26]. *Trichoderma* species are well-known for their capability to secrete a large number of secondary metabolites, such as plant growth promoters and a variety of enzymes [27]. The fungus genus *Trichoderma* is one of the most investigated groups of fungi utilized as biological control agents [28]. *Trichoderma* species are known to produce antibiotics or low-molecular-weight compounds [29]. Fungi, particularly *Trichoderma* species, are known to produce metabolites with antibacterial, anticancer, antioxidant, and antifungal properties among microbes. *Trichoderma* has been studied extensively as a biocontrol agent, biofungicide, biofertilizer, and plant growth enhancer. However, research into the medicinal potential of *Trichoderma* metabolites has received limited attention [30]. Therefore, in this study, ZnO NPs were synthesized from *Trichoderma* spp.

Traditional antimicrobial or antibiotic therapies used for bacterial diseases rely on the use of antimicrobial compounds or antibiotics that can inhibit or destroy microbial cell development. Pathogenic microbes, however, can build biofilms to defend themselves against inhibitory chemicals [31]. A colony of bacteria living in a self-produced matrix of biopolymers adhered to surfaces is referred to as a “biofilm”. Microbes prefer to form biofilms on surfaces, avoiding the detrimental effects of antibiotics and detergents, and they persist in hospitals, generating a high number of hospital-acquired diseases [32]. Given that *S. aureus* is found in the skin’s natural flora, it is likely that it is one of the most prevalent causal agents in hospital-acquired infections involving medical implants [33]. Furthermore, *S. aureus* has been shown to be resistant to larger dosages of antibiotics, perhaps contributing to the development of antibiotic-resistant insusceptible strains [34]. Based on our previous study [35], *Trichoderma* spp. isolated from rhizosphere soil was chosen for the biosynthesis of ZnO NPs and to determine their antibacterial activity against human pathogens. The present study is focused on the biosynthesis of ZnO NPs from *Trichoderma* spp. and their antibacterial action against human pathogens *S. aureus* and *E. coli,* as well as the inhibition of biofilm formation (*S. aureus*) by using different dosages of ZnO NPs.

## 2. Materials and Methods

### 2.1. Collection of Rhizosphere Soil

For this study, 10 g of rhizosphere soil was collected by uprooting a plant. The soil samples were collected and stored in polythene bags at 4 °C until further use [36].

### 2.2. Isolation and Identification of Fungi from Rhizosphere Soil

The collected rhizosphere soil sample was serially diluted into different concentrations and vortexed well. The supernatants were then transferred to sterilized potato dextrose agar (PDA) medium obtained from Himedia, India, and they were incubated for 7 days at 26 ± 2 °C on plates with the standard antibiotic chloramphenicol. Following incubation, the fungal colonies obtained on the PDA plates were isolated and re-inoculated into freshly prepared sterile plates. The obtained single-spore colonies were subjected to morphological and molecular characterization procedures after a 7-day incubation period [37]. For the isolation of genomic DNA from the fungi, a DNA isolation kit was procured from Chromous Biotech, India, and it was used by following the method described in the manufacturer’s manual. 18S rRNA gene amplification was carried out using PCR, and the obtained gene sequence of the test strain was compared against the nucleotide collection (nr/nt) database using the BLAST program [38,39]. The gene sequences were deposited in GenBank, and the following accession number was obtained: OL826855. A similar study was carried out, where medicinal plants were used against *Streptococcus pyogenes* [40].

### 2.3. Green Synthesis of Zinc Oxide Nanoparticles (ZnO NPs)

In the present study, a green synthesis protocol was used for the synthesis of ZnO NPs from the rhizosphere soil fungus *T. asperellum*. The substrate zinc nitrate hexahydrate (Zn (NO_3_)2. 6H_2_O) was purchased from Sigma-Aldrich (analytical grade), and it was used for the synthesis of ZnO NPs without any further purification. The substrate solutions were prepared by dissolving 1 g of Zn (NO_3_)2.6H_2_O in 10 mL of double-distilled water, then adding 2 mL of a fungal extract of *T. asperellum*, and stirring the reaction mixture for ~5−10 min using a magnetic stirrer. A preheated muffle furnace, maintained at 400 ± 10 °C, was used where the obtained mixture was stored, and at that temperature, the reaction mixture boils, bubbles, and foam dehydrates in less than 3 min. The resulting product was calcinated for 2 h at 700 °C, and the final obtained product was used for further studies [41,42].

### 2.4. Characterization of Zinc Oxide Nanoparticles (ZnO NPs)

The formation of ZnO NPs was confirmed by recording UV–visible absorption spectra with a UV–visible spectrophotometer (SL 159 ELICO). The chemical composition and surface functional groups of the sample were evaluated by using Fourier transform infrared spectroscopy (FTIR) (Varian 3100). A powder X-ray diffractometer (PXRD) (Shimadzu) with Cu Kα (1.5418 Å) radiation and a nickel filter was used to examine the phase purity and crystalline nature of the ZnO NPs. The shape and surface morphology of the synthesized nanoparticles were analyzed by using scanning electron microscopy (SEM) (Hitachi Tabletop TM-3000) and an energy-dispersive analysis of X-rays (EDAX). To determine the size of the synthesized nanoparticles, high-resolution transmission electron microscopy (HRTEM) (JEOL JEM 2100), along with selected area electron diffraction (SAED), was used [43].

### 2.5. In Vitro Screening of ZnO NPs for Their Antibacterial Property against Human Pathogens

The pathogenic cultures of *S. aureus* (NCIM No. 2079) and *E. coli* (NCIM No. 2556) used in this study were National Collection of Industrial Microorganisms (NCIM) cultures. The antibacterial property of biogenic ZnO NPs against the selected pathogens was assessed by using the disc diffusion method. The standard 0.5 McFarland concentration of the selected bacterial culture was used to prepare a culture on Mueller–Hinton agar medium plates (procured from Himedia, India) using sterile swabs. Different concentrations of biogenic ZnO NPs (25, 50, and 75 µg/mL) were loaded onto the sterile discs. The test plate was placed with a positive control disc, a negative control disc, and biologically synthesized ZnO NPs. The standard antibiotic tetracycline (100 µg/mL) was used as a positive control, and sterile distilled water was used as a negative control before the plates were incubated at 28 ± 2 °C for 24 h. After incubation, the plates were examined for the inhibition zone, and the results were recorded in mm [44].

To determine the minimum inhibitory concentration (MIC) of the biosynthesized ZnO NPs against the selected pathogens, the broth microdilution protocol was followed with minor modifications. Different ZnO NP concentrations were prepared by diluting the stock solution of the ZnO NPs (1 mg/mL) with the dilution in the Mueller–Hinton Broth (MHB) medium and then by loading them onto sterile 96-well microtiter plates. To each well of the microtiter plate, 10 µL of bacterial suspension was added and incubated for 24 h at 28 °C. MHB was kept as a negative control, and the standard tetracycline at a concentration of 100 µg/mL was used as a positive control; all experimental tests were performed in triplicate. After 24 h of incubation, 20 µL of iodonitrotetrazolium chloride dye (INT) (0.5 µg/mL) was added to each well, followed by the incubation of the microtiter plates at 28 °C for 60 min. The MIC value indicates the sample concentration that prevents the color shift from colorless to red, where the colorless tetrazolium salt works as an electron acceptor and is reduced by active organisms to a red-colored formazan product [45].

### 2.6. Fluorescence Microscopy and Scanning Electron Microscopic Analysis

The biosynthesized ZnO NPs at a concentration of 75 µg/mL showed promising antibacterial activity. The dead and live cells of the treated *E. coli* and *S. aureus* were monitored by using fluorescent microscopy. The pathogenic bacteria *E. coli* and *S. aureus* were treated with the ZnO NPs (75 µg/mL) and incubated for 24 h at 37 °C. After incubation, the bacteria were stained with 1 µL of ethidium bromide and acridine orange, and then they were incubated in the dark for 10–15 min. The nuclei of the bacterial cells were stained green with acridine orange procured from Thermofisher, US, whereas the nuclei of the bacterial cells were stained orange with ethidium bromide procured from Thermofisher, US. Then, 10 μL of each of the test strains was put on a slide and viewed under a fluorescent microscope (Carl Zeiss, barrier filter O 515, excitation filter BP 490) at 40X magnification. SEM microscopy was used to investigate the morphological properties of the ZnO-NP-treated bacterial cells [43].

### 2.7. Antiadherence Assay

Inoculate 50 μL of the ZnO NPs at various concentrations (25, 50, and 75 μg/mL) into selected wells. From a 24 h bacterial culture, make a bacterial suspension (1 × 10^8^ CFU/mL) in 15 mL. To obtain a 10^6^ CFU/mL bacterial suspension, carry out a 1:100 dilution in a separate centrifuge tube. Using a suitable pipette, add 50 μL of the diluted bacterial concentration to the relevant wells. In this study, the organism was only permitted to grow without the ZnO NPs in the control set. To avoid the evaporation of the water from the test wells, fill the neighboring wells of the microplate with sterile distilled water. Water evaporation in the test wells might tamper with the results. Place the plate in an incubator at 37 °C for 18 to 24 h, covered with the lid. Remove the 96-well plate from the incubator and slowly decant or pipette the nutrient broth (NB). Allow the plate to air dry beneath the biosafety cabinet after rinsing it three times with sterile, double-distilled water. Turn the plates upside down to speed up the drying process. Before moving onto the next stage, make sure that they are completely dry. Fill the test wells with 100 mL of aqueous crystal violet (1 percent *w/v*), and let it stain the bacterial cell walls for 10 to 15 min. Into a sink, decant the crystal violet. Allow the test wells to dry beneath the biosafety cabinet after three rinses with sterile, double-distilled water. To solubilize the crystal violet, dissolve 30 % (*v/v*) glacial acid in water, and let it stand for 15 min. In each of the test wells, make sure that there is a clear blue/violet solution with no apparent residue. Examine the UV absorbance at 570 nm of each well. Using the formula below, calculate the antiadherence activity of the ZnO NPs and tetracycline [45,46].

$$\mathrm{Antiadherence}\;\mathrm{activity}\;\%=\frac{\mathrm{Absorbance}\;\mathrm{of}\;\mathrm{control}-\mathrm{Absorbance}\;\mathrm{of}\;\mathrm{ZnO}\;\mathrm{NPs}}{\mathrm{Absorbance}\;\mathrm{of}\;\mathrm{control}}\;\times \;100$$

### 2.8. Antibiofilm Assay

To make a 10^6^ CFU/mL bacterial suspension, follow procedures similar to those of the antiadherence assay. In a fresh 96-well microplate, inoculate 100 μL of the diluted bacterial suspension in NB into each well and incubate the plate for 24 h at 37 °C. Remove all of the NB broth from the microplate, and wash the wells three times with sterile phosphate-buffered saline (PBS) to avoid damaging the biomass growing on the bottom and the walls of the wells. In this study, we use 100 μL of sterile NB as a control, the ZnO NPs suspended in NB with the test concentrations of 25, 50, and 75 μg/mL, and NB containing a concentration of tetracycline of 100 μg/mL. Remove the 96-well plate from the incubator, and slowly decant or pipette the NB. Allow the plate to air dry beneath a biosafety cabinet after rinsing it three times with sterile, double-distilled water. Turn the plates upside down to speed up the drying process. Before moving on to the next stage, make sure that they are completely dry. Fill the test wells with 100 mL of aqueous crystal violet (1% *w/v*), let it stain the bacterial cell walls for 10 to 15 min, and then decant the crystal violet. Allow the test wells to dry beneath the biosafety cabinet after three rinses with sterile, double-distilled water. To solubilize the crystal violet, dissolve 30% (*v/v*) glacial acid in the water, and allow it to stand for 15 min. In each of the test wells, make sure that there is a clear blue/violet solution with no apparent residue. The UV-spec absorbance at 570 nm of each well was examined. Using the formula below, calculate the antibiofilm activity of the test material and tetracycline [47,48,49,50].

$$Antibiofilm\;\%=\frac{Absorbance\;of\;control-Absorbance\;of\;ZnONPs}{Absorbance\;of\;control}\;\times \;100$$

### 2.9. Microscopic Studies

#### 2.9.1. Bright-Field Microscopic Studies

After biofilm formation, the Gram-stained sample of S. aureus was examined under a bright-field microscope with a 100X objective in an oil immersion medium. Similarly, the samples with the ZnO NPs and tetracycline treatment were examined under a microscope [51].

#### 2.9.2. Scanning Electron Microscopy (SEM)

The biofilm morphology was studied using a modified SEM approach. The test samples were fixed in 3% glutaraldehyde at 4–6 °C for 24 h. At each interval, the cells were washed three times with 0.1 M PBS for 10 min each time. The cultures were dehydrated for 10 min in the different gradient alcohol concentrations (50–100%). To keep the specimen from drying out, it was placed in 100% alcohol and attached to an aluminum stub with carbon tape before being sputter-coated with gold [52].

### 2.10. Statistical Analysis

All the obtained results of the antibacterial experiments were analyzed statistically using SPSS software (version 20.0) and Microsoft Excel.

## 3. Results and Discussions

### 3.1. Collection, Isolation, and Identification of Fungi from Rhizosphere Soil

The rhizosphere soil sample was collected from Eleusine coracana (Ragi) in Ramanagara district, southern Karnataka. Only *Trichoderma* spp. were isolated from the collected rhizosphere soil. The fungal colonies were grown on potato dextrose agar (PDA) medium plates supplemented with antibiotics, and the plates were incubated at 28 °C for 6–7 days. The isolated fungi were identified based on cultural and colony characteristics and a microscopic observation by using standard manuals Barnett and Hunter [53]; based on this, the fungi were identified as *Trichoderma* spp.

### 3.2. Molecular Characterization of Fungi Isolated from Rhizosphere Soil

The fungi isolated from Eleusine coracana (Ragi) were subjected to molecular characterization, which was validated by database searches using BLAST tools at the National Centre for Biotechnology Information (Bethesda, MD, USA); this revealed the fungi to be *Trichoderma asperellum* with a 99% similarity. Similar studies were conducted by Tomah et al. [29]; in this report, *Trichoderma* spp. were isolated from the soil sample and identified by using molecular methods.

### 3.3. Characterization of Biosynthesized Zinc Oxide Nanoparticles (ZnO NPs)

**Figure 1:**
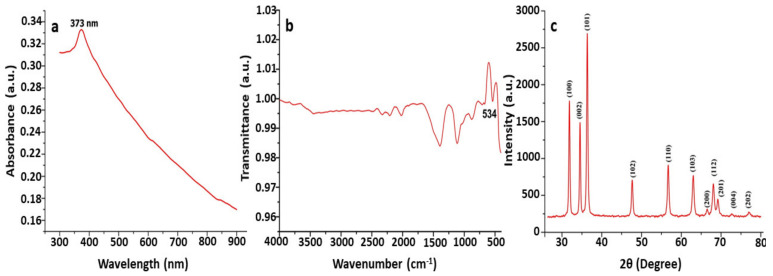
Characterization of biosynthesized ZnO NPs using *T. asperellum*: (**a**) UV—vis spectra; (**b**) FT-IR spectrogram; (**c**) PXRD patterns.

UV–Vis spectroscopy is a commonly used approach for the characterization and confirmation of synthesized ZnO NPs based on surface plasmon resonance (SPR) peaks. A characteristic absorption peak was observed at 373 nm by using UV–Vis spectroscopy for the synthesized ZnO nanoparticle suspension. The reported absorption peaks of the ZnO NPs are in good accordance with those in previous research, where absorption peaks have been found between 355 and 380 nm. Our results agree with those of Shaikhaldein et al. [54], who obtained an absorption peak at 380 nm. In a study conducted by Mahamuni et al. [55], the absorption peak recorded in each spectrum was found to be in the range of 360–380 nm, which is a characteristic feature of pure ZnO. The study conducted by Wang et al. [56] obtained a maximum peak at 330 nm, confirming that the synthesized nanoparticles in this study were ZnO NPs (Figure 1).

The application of the FTIR approach in the analyses of biosynthesized ZnO NPs has been useful in identifying the biomolecules involved in the formation of ZnO NPs [57]. The absorbance was recorded at 400 cm^−1^ to 600 cm^−1^ in the FTIR spectrum, confirming that ZnO nanoparticles were synthesized using *T. asperellum* (Figure 1). Our results agree with the results of Pillai et al. [58], who found a noticeable peak at 482 cm^−1^, confirming the presence of ZnO NPs in this study. In a study conducted by Selim et al. [59], the FTIR spectra showed a peak at 442 cm^−1^ for biosynthesized ZnO NPs. Jayappa et al. [60] reported that absorption peaks found at 475 cm^−1^, 486 cm^−1^, and 473 cm^−1^ depict the inter-atomic vibrations that cause stretching vibrations in metallic ZnO (Figure 1).

ZnO NPs are subjected to intense rays from XRD machines during XRD examinations, and these rays pierce through the ZnO NPs and offer crucial details about their structure [55]. The PXRD graph’s unique peaks confirmed and validated the biosynthesized ZnO nanostructure with the *Trichoderma* spp. fungus extract. The obtained PXRD patterns of the biosynthesized ZnO NPs revealed the presence of distinct peaks and are well-matched with JCPDS No. 89-7102. The crystalline structure of the synthesized ZnO NPs was observed to have stiff and narrow diffraction peaks, with no substantial variations in the diffraction peaks, indicating that the crystalline product was impurity-free. Peaks were absent from other phases or contaminants, indicating that the product was pure-phase ZnO NPs. Scherrer’s formula was applied to the first intense PXRD peaks, and the sizes of the green synthesized ZnO NPs were found to be between 44 nm and 78 nm. The XRD peaks found in this work are comparable to the XRD patterns previously reported for ZnO nanoparticles produced using *Xylaria acuta*. The results are equivalent to those of nanoparticles produced with a crystallographic hexagonal wurtzite structure [61] (Figure 1).

D = 0.9 × λ/(β cosθ)

where λ is the wavelength of the X-ray (1.542 Å), β refers to the full width at half maximum (FWHM in radian) caused by the crystallites, and θ refers to the Bragg angle.

**Figure 2:**
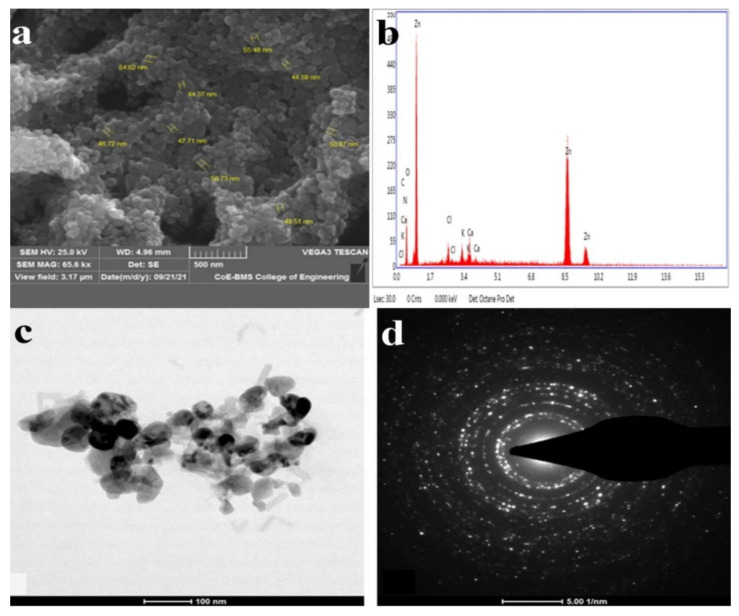
(**a**) High-magnification SEM image of ZnO NPs synthesized from *T. asperellum*: (**b**) energy-dispersive X-ray spectroscopy (EDAX) analysis; (**c**) TEM images of ZnO NPs synthesized from *T. asperellum*; and (**d**) SAED pattern of ZnO NPs synthesized from *T. asperellum*.

Signals were created and recorded by the detector when the ZnO NPs were subjected to electron beams. Information on the shape, orientation, and crystalline structure of ZnO NPs may be derived from the recorded signal [56]. The surface morphologies of the ZnO NPs synthesized by *T. asperellum* were recorded at different magnifications and depicted in SEM images, which revealed the form and size of the zinc oxide nanoparticles. The SEM micrographs revealed a variety of nanoparticle combinations, as well as unique ZnO NPs. The produced forms of the ZnO NPs with various surface morphologies were also demonstrated using SEM images. An EDAX analysis was used to determine the qualitative and quantitative differences between the materials involved in the making of the nanoparticles. The synthesized nanoparticles were found to have the greatest proportions of zinc and oxygen in the analysis. The additional elements, as well as zinc, were detected in the synthesized ZnO NPs using an EDAX spectrum. The presence of metallic zinc oxide in the biosynthesized ZnO NPs was confirmed by the EDAX analysis. Our results are in good agreement with the results obtained by Shobha et al. [35], indicating that the biologically synthesized ZnO NPs had a similar elemental proportion of zinc and oxygen (Figure 2).

The TEM images revealed agglomerated ZnO NPs that were composed of well-dispersed minute particles. The size of the synthesized ZnO NPs ranged from 44 nm to 78 nm, according to the TEM investigation. The HRTEM and SAED patterns were found to correspond to ZnO compounds. The TEM picture depicts agglomerated, tiny ZnO NPs. Well-defined crystal planes can be seen in the high-resolution TEM picture. The particle size of the biosynthesized ZnO NPs from *T. asperellum* ranged from 44 nm to 78 nm. The (hkl) values corresponding to the significant peaks in the PXRD profiles fit the SAED patterns very well [60] (Figure 2).

### 3.4. In Vitro Screening of Zinc Oxide Nanoparticles (ZnO NPs) for Their Antibacterial Activity against Human Pathogens

A wide variety of metabolites, such as alkaloids, terpenoids, and flavonoids, are known to be produced by fungi and exhibit various properties, such as antibacterial, antiviral, anti-inflammatory, antitumor, and antifungal properties [62]. In a similar study, gold nanoparticles were synthesized from Bauhinia tomentosa Linn leaf extracts and tested for antibacterial activity against *E. coli* and *S. aureus* [63]. In the present study, *S. aureus* and *E. coli* were used to examine the antibacterial activity of ZnO NPs. By measuring the inhibition zone surrounding the disc, the antibacterial activity of the ZnO NPs was studied. The disc diffusion approach was used to investigate the antibacterial activity of the biosynthesized ZnO NPs, where the discs were placed on Mueller–Hinton agar (MHA) medium plates that were pre-swabbed with the bacteria. The inhibition zone was measured and tabulated. The MIC values of the biosynthesized ZnO NPs were determined using the 96-well microplate technique. Because of their smaller size and high surface-to-volume ratio, ZnO NPs have considerable antibacterial activity [61]. The current work clearly shows that ZnO NPs may be used as antibacterial agents against the human pathogens *S. aureus* and *E. coli* (Table 1).

**Figure 3:**
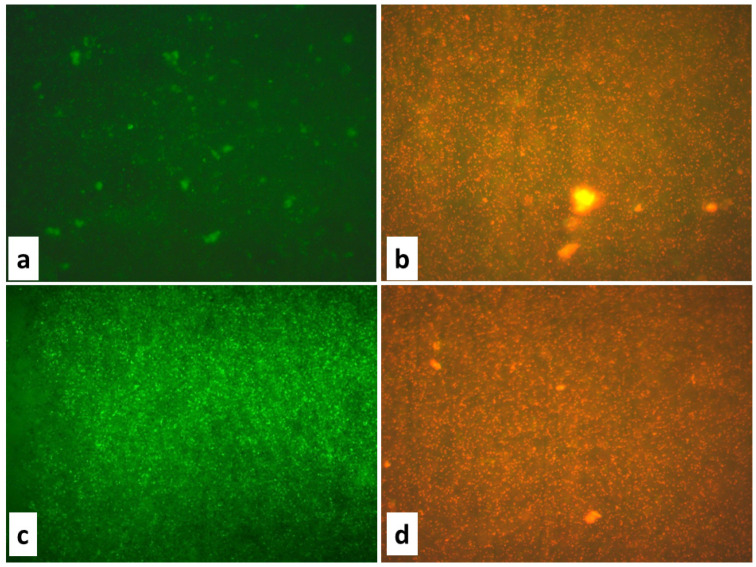
Fluorescence microscopic images of (**a**) *E. coli* control, (**b**) *E. coli* treated with ZnO NPs at a concentration of 75 μg/mL, (**c**) *S. aureus* control, (**d**) *S. aureus* treated with ZnO NPs at a concentration of 75 μg/mL.

The antibacterial activity of the biosynthesized ZnO NPs was further confirmed using fluorescence microscopy. The control bacterial cells were green under fluorescence microscopic inspection, which is a marker of healthy morphology in microorganisms, but the ZnO-NP-treated bacterial cells were red, with noticeable modifications in the cell wall, such as collapse, shrinkage, and a non-homologous surface. The absence of ZnO NPs (control microbial cells) resulted in the normal shape of microbial cells, as shown in the micrographs. However, the bacterial cells treated with the ZnO NPs showed morphology changes, such as ruptured cell membranes, oozed-out contents, and the aggregation of cells. Scanning electron microscopy (SEM) studies were undertaken to develop a better understanding of how the ZnO NPs cleaved the bacterial cell membrane and to predict the mechanism of the cell membrane rupture caused by the ZnO NPs. Furthermore, the effect of the ZnO NPs on the bacterial cells in comparison to the untreated bacterial cells was assessed by using SEM. The absence of ZnO NPs (control bacterial cells) resulted in the normal shape of bacterial cells, as seen in the micrographs. The bacterial cells treated with the ZnO NPs, however, displayed morphological changes, with the cell membrane rupturing, contents oozing out, and cells clumping together (Figure 3).

### 3.5. Determination of Biofilm Formation

**Figure 4:**
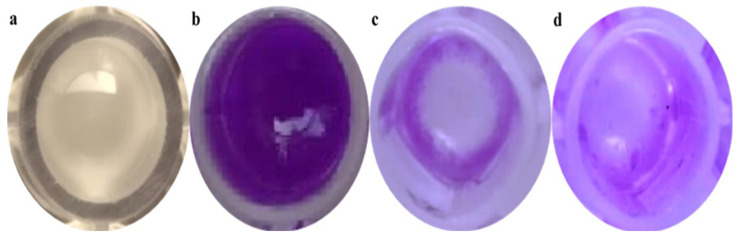
Crystal violet assay carried out to determine the antibiofilm activity of samples against *S. aureus* biofilms. (**a**) *S. aureus* forming a biofilm after 24 h of incubation in a microtiter well, (**b**) a well after crystal violet staining, (**c**) a control (tetracycline) well containing the crystal violet biofilm, and (**d**) a well with ZnO NPs (75 μg/mL) after decanting the crystal violet reduction of biofilms.

The violet stains showed biofilm development according to the methodology. This indicates that the NB medium was enough for biofilm formation, whereas a 0.2% (*w/v*) crystal violet concentration was suitable for a naked-eye inspection and spectrophotometer measurement (Figure 4). The biosynthesized ZnO NPs were proved to have efficient activity against planktonic bacteria using the minimum inhibitory concentration (MIC) assay. The affinity of the ZnO NPs for planktonic microorganisms was further investigated. The particles’ antibiofilm effectiveness was tested against Gram-positive microorganisms (*S. aureus*).

### 3.6. Assessment of Antiadherence Assay

**Figure 5:**
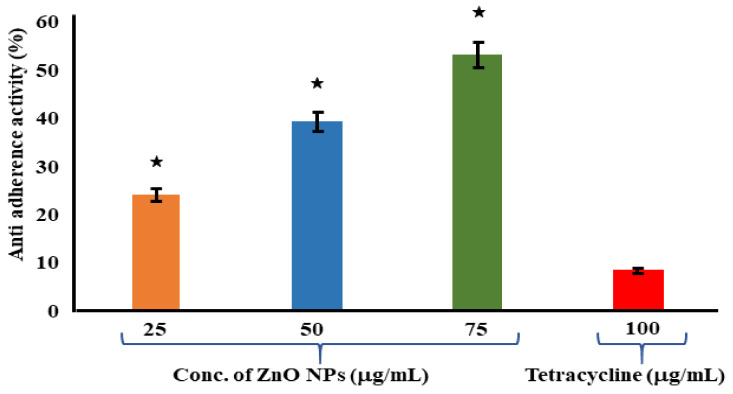
Antiadherence assay using tetracycline as positive control and *S. aureus* as growth control. The experiment was evaluated based on triplicate results with standard deviation (*n* = 3, *p* < 0.05). * Indicates a significant difference when compared to the negative control (NB only).

The antiadherence properties of ZnO NPs and tetracycline has been determined by using an antiadherence assay, which uses a 96-well microplate. The biomass of the bacterial cell was quantified using a microplate reader at 570 nm, revealing a declining trend in biomass attachment as the quantity of the examined ZnO NPs increased. According to the findings, the ZnO NPs at 75 μg/mL had a strong antiadherence activity of 53.24 ± 1.37%. When compared to the control, the tetracycline at 100 μg/mL had no significant antiadherence action (Figure 5). The crystal violet staining of the biofilms was used to test the adherence of the biofilm bacteria on the microplates in order to determine whether the ZnO NPs inhibited biofilm development. At 25 μg/mL of ZnO NPs, biofilm development was dramatically reduced. The ZnO-NP-induced suppression of *S. mutans* on the oral surface has been recently established in research [64]. A similar study was carried out, where eugenol was investigated for its inhibitory property against the adherence and biofilm formation of *Streptococcus mutans* [65]. In another previous study, aquatic extracts of *Viscus album* and *Apium graveolens* showed antibiofilm and antiadherence against clinical bacterial isolates [66].

### 3.7. Assessment of Antibiofilm Assay

**Figure 6:**
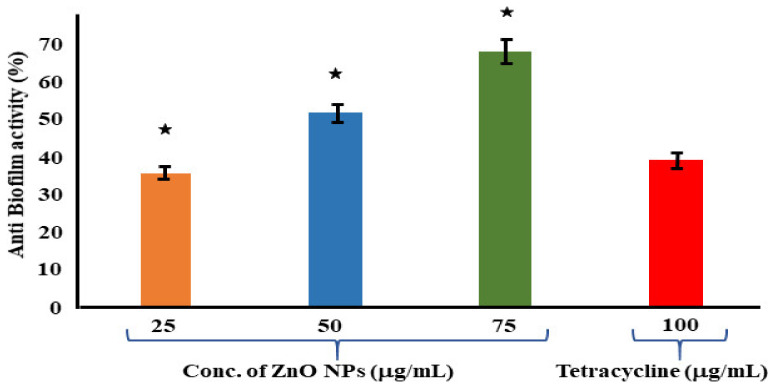
Antibiofilm assay using tetracycline as positive control and *S. aureus* as growth control. The experimental results were evaluated based on triplicate results with standard deviation (*n* = 3, *p* < 0.05). * Indicates a significant difference when compared to the negative control (NB only).

The antibiofilm properties of the ZnO NPs and tetracycline were determined using a 96-well microplate. When compared to the control (NB alone), the biomass of the bacterial cells was quantitatively examined on a microplate reader at an absorbance of 570 nm. The results revealed a considerable reduction in biofilm formation with an increase in the quantity of the biologically synthesized ZnO NPs. According to the results, the ZnO NPs at 75 μg/mL had a significant antibiofilm activity of 68.46 ± 1.72%. Meanwhile, the antibiofilm activity of the positive control tetracycline at a greater concentration of 100 μg/mL was measured to be 39.23 ± 4.61% (Figure 6). Reactive oxygen species (ROS) formation takes place when foreign particles come into contact with a bacterial solution, and this plays a crucial role in bacterial inhibition. Biofilm formation inhibition was investigated in planktonic *S. aureus* exposed to reagents for 24 h at the start. The ZnO NPs were more efficient than the tetracycline in eradicating the premade biofilm produced by *S. aureus*, according to a conventional crystal violet test for biofilm biomass. A similar study was conducted on *S. mutans*, where a solvent fraction of *Trachyspermum ammi* was used to evaluate the expressions of the genes involved in biofilm formation [67].

### 3.8. Microscopic Studies

**Figure 7:**
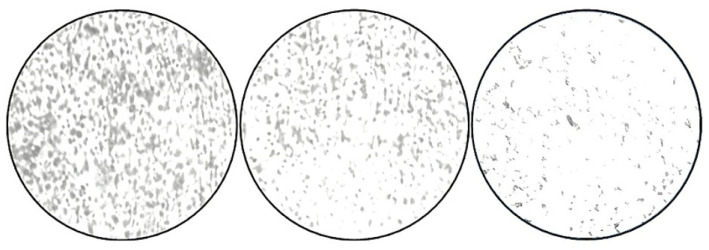
Micrographs of *S. aureus* biofilms. Adherence of *S. aureus* onto the coverslips; control (NB alone), tetracycline, and ZnO nanoparticles (75 μg/mL), as examined by CV staining.

The bright-field compound microscopic photographs revealed a decrease in the cells when they were Gram-stained, based on microscopic examinations. When compared to the negative control (NB alone), the positive control (tetracycline-treated) and the ZnO-NP-treated wells showed a lower number of cells (Figure 7).

**Figure 8:**
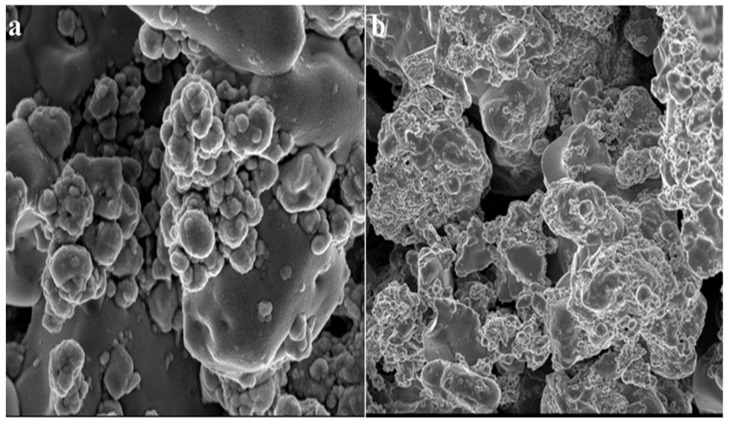
SEM micrographs of biofilm mass: (**a**) zinc oxide nanoparticles attached to the *S. aureus* biofilm, (**b**) biofilm of *S. aureus* disturbed after 24 h of treatment with ZnO NPs.

After 24 h of incubation, SEM micrographs of the tested bacterium, *S. aureus,* treated with the ZnO NPs were taken. The SEM micrographs assisted in elucidating the mechanism/relationship between the bacteria and the ZnO NPs, as well as their antibacterial activity (Figure 7). In the case of *S. aureus*, it is obvious from the micrographs that the ZnO NPs initially adhered to the cell’s outer membrane and then penetrated the cell entirely, perhaps resulting in cell death. The use of SEM to visualize the bacterial biofilms revealed a wide range of morphological changes in the biofilm topologies (Figure 7 and Figure 8). There were remarkably fewer dispersed cell aggregates and fewer viable cells in the aggregates in the biofilms after 24 h of exposure to the ZnO NPs. The ZnO NPs were shown to be superior in inhibiting *S. aureus* biofilm formation when the biofilm biomass was measured [68,69]. These findings show that the ZnO NPs were more efficient than the tetracycline in inhibiting biofilm development and in disrupting the preformed biofilms of *S. aureus.*

## 4. Conclusions

The present study highlights the biosynthesis of ZnO NPs from *T. asperellum*. The synthesized nanoparticles were characterized by UV–visible spectroscopy, PXRD, FTIR, SEM with EDAX, and TEM with SEAD patterns. This research focused on the biological synthesis of ZnO NPs, which is both cost-effective and environmentally friendly. Nanosized particles are more efficient in inhibiting the growth of microbes. Our studies emphasize that biologically synthesized ZnO NPs can be used as efficient antimicrobial agents in various fields, as ZnO NPs are non-toxic; have antimicrobial, barrier, and mechanical properties; and belong to the Generally Recognized as Safe (GRAS) category. Additionally, the non-central symmetry and biocompatible nature of ZnO make it the most important nanomaterial in research and applications. Based on our findings, we can conclude that the use of ZnO NPs is effective against the vast majority of pathogenic bacteria and biofilm-producing bacteria. ZnO NPs can be used for surgical instruments, which usually become colonized by bacterial biofilms. The future prospects of this study are to check the stability of these nanoparticles and their mode of action at the molecular level.

## Figures and Tables

**Table 1:** ZnO NP antibacterial activity against *S. aureus* and *E. coli*.

Concentrations of ZnO NPs (μg/mL)	Disc Diffusion Values (mm)		MIC Values (µg/mL)	
	*S. aureus*	*E. coli*	*S. aureus*	*E. coli*
25	3.18 ± 0.12	2.52 ± 0.49	25	50
50	6.23 ± 0.42	5.69 ± 0.38	12.5	25
75	9.82 ± 0.73	7.37 ± 0.27	6.25	12.5
Positive 100 μg/mL	8.37 ± 0.12	6.14 ± 0.19	50	50
Negative	0	0	0	0

## Data Availability

Data is available in this manuscript.
