# Correction: Shobha et al. Trichoderma-Mediated ZnO Nanoparticles and Their Antibiofilm and Antibacterial Activities. *J. Fungi* 2023, *9*, 133

**DOI:** 10.3390/jof11040304

**Published:** 2025-04-11

**Authors:** Balagangadharaswamy Shobha, Bagepalli Shivaram Ashwini, Mohammed Ghazwani, Umme Hani, Banan Atwah, Maryam S. Alhumaidi, Sumanth Basavaraju, Srinivas Chowdappa, Tekupalli Ravikiran, Shadma Wahab, Wasim Ahmad, Thimappa Ramachandrappa Lakshmeesha, Mohammad Azam Ansari

**Affiliations:** 1 Department of Microbiology and Biotechnology, Bangalore University, Jnana Bharathi Campus, Bengaluru 560056, India; 2 Department of Microbiology, Sri Siddhartha Medical College, Tumkur 572107, India; 3 Department of Pharmaceutics, College of Pharmacy, King Khalid University, Abha 62529, Saudi Arabia; 4 Laboratory Medicine Department, Faculty of Applied Medical Sciences, Umm Al-Qura University, Makkah 24382, Saudi Arabia; 5 Department of Biology, College of Science, University of Hafr Al Batin, Hafr Al Batin 31991, Saudi Arabia; 6 Department of Pharmacognosy, College of Pharmacy, King Khalid University, Abha 61421, Saudi Arabia; 7 Department of Pharmacy, Mohammed Al-Mana College for Medical Sciences, Dammam 34222, Saudi Arabia; 8 Department of Epidemic Disease Research, Institute for Research and Medical Consultations (IRMC), Imam Abdulrahman Bin Faisal University, Dammam 31441, Saudi Arabia

## Error in Figures

In the original article [1], the wrong “Figures 3 and 7” were uploaded due to the author’s negligence as published. The authors of the published paper [1] would like to correct Figures 3 and 7. The corrected “Figure 3 and Figure 7” appear below. The authors apologize for any inconvenience caused. The authors state that the scientific conclusions are unaffected. This correction was approved by the Academic Editor. The original article has also been updated.

## Figures and Tables

**Figure 3:**
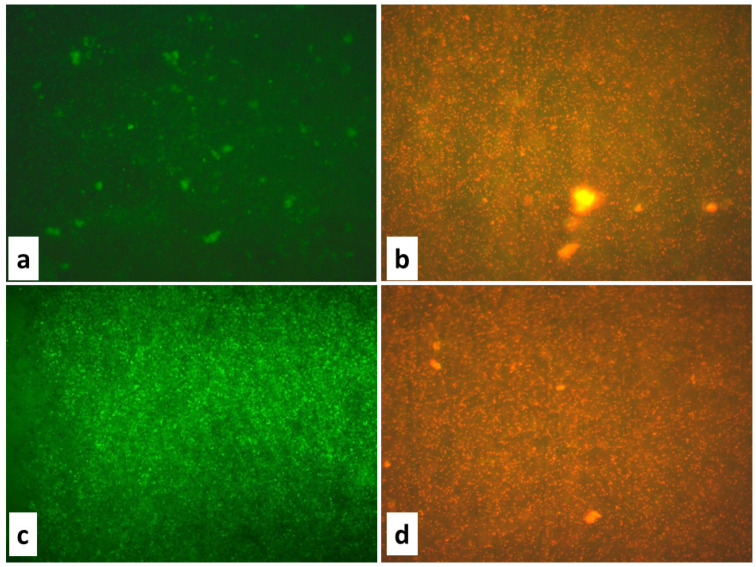
Fluorescence microscopic images of (**a**) *E. coli* control, (**b**) *E. coli* treated with ZnO NPs at a concentration of 75 μg/mL, (**c**) *S. aureus* control, (**d**) *S. aureus* treated with ZnO NPs at a concentration of 75 μg/mL.

**Figure 7:**
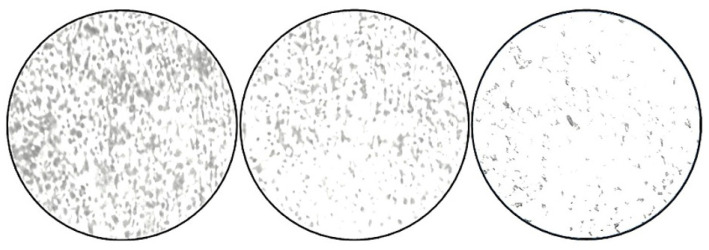
Micrographs of *S. aureus* biofilms. Adherence of *S. aureus* onto the coverslips; control (NB alone), tetracycline, and ZnO nanoparticles (75 μg/mL), as examined by CV staining.
